# Construction of a Genetic Prognostic Model in the Glioblastoma Tumor Microenvironment

**DOI:** 10.3390/genes16080861

**Published:** 2025-07-24

**Authors:** Wenhui Wu, Wenhao Liu, Zhonghua Liu, Xin Li

**Affiliations:** 1 College of Life Science, Northeast Agricultural University, Harbin 150030, China; s220901080@neau.edu.cn (W.W.); b230901013@neau.edu.cn (W.L.); 2 State Key Laboratory of Organ Regeneration and Reconstruction, Institute of Zoology, Chinese Academy of Sciences, Beijing 100101, China; 3 Beijing Institute for Stem Cell and Regenerative Medicine, Beijing 100101, China

**Keywords:** glioblastoma, tumor microenvironment, nomogram, prognosis

## Abstract

Background: Glioblastoma (GBM) is one of the most challenging malignancies in all of neoplasms. These malignancies are associated with unfavorable clinical outcomes and significantly compromised patient wellbeing. The immunological landscape within the tumor microenvironment (TME) plays a critical role in determining GBM prognosis. By mining data from The Cancer Genome Atlas (TCGA) and Gene Expression Omnibus (GEO) databases and correlating them with immune responses in the TME, genes associated with the immune microenvironment with potential prognostic value were obtained. Method**:** We selected GSE16011 as the training set. Gene expression profiles were substrates scored by both ESTIMATE and xCell, and immune cell subpopulations in GBM were analyzed by CIBERSORT. Gene expression profiles associated with low immune scores were performed by lasso regression, Cox analysis and random forest (RF) to identify a prognostic model for the multiple genes associated with immune infiltration in GBM. Then we constructed a nomogram to optimize the prognostic model using GSE7696 and TCGA-GBM as validation sets and evaluated these data for gene mutation and gene enrichment analysis. Result: The prognostic correlation between the six genes *(MEOX2*, *PHYHIP*, *RBBP8*, *ST18*, *TCF12,* and *THRB)* and GBM was finally found by lasso regression, Cox regression, and RF, and the online database obtained that all six genes were differentially expressed in GBM. Therefore, a prognostic correlation model was constructed based on the six genes. Kaplan–Meier (KM) survival analysis showed that this prognostic model had excellent prognostic ability. Conclusions: Prognostic models based on tumor microenvironment and immune score stratification and the construction of related genes have potential applications for prognostic analysis of GBM patients.

## 1. Introduction

Global incidence of central nervous system (CNS) malignancies demonstrated a sustained increase between 1990 and 2016. In 2016, brain and CNS tumors affected roughly 330,000 individuals, resulting in 227,000 fatalities [1]. GBM, a CNS tumor, represents the most common primary malignant brain cancer [2,3]. GBM currently has multiple therapeutic approaches, yet clinical outcomes remain unfavorable, demonstrating merely a 5.5% five-year survival probability [4,5]. Consequently, identification of potential GBM biomarkers through clinical expression profiling represents a critical strategy for prognostic improvement, personalized treatment optimization, and patient survival enhancement.

The patient’s tumor tissue comprises not only neoplastic cells but also various host-derived components, including cancer-associated stromal cells and immune populations [6,7]. Dynamic tumor–microenvironment interactions exhibit stage-specific effects on cancer progression, ranging from growth promotion to suppression during tumorigenesis and metastasis [8,9]. TME-resident immune populations mediate malignant cell clearance via multiple immunosurveillance pathways [10,11]. However, cancer cells evade immune surveillance through immunosuppressive strategies, including recruitment of regulatory immune cells and reduction of tumor antigen presentation [12,13]. With the widespread application of gene expression profiling in oncology, prognostic modeling for cancers has advanced significantly [14,15]. Cancer-linked healthy cell invasion impacts predictive modeling via gene expression data [16,17]. Computational methods have been developed to quantify tumor cell proportions within the tumor microenvironment, with immune and stromal cells being critical for prognostic modeling [18]. Bioinformatic approaches have been established to quantify tumor composition using transcriptomic data from public repositories. A representative example is the ESTIMATE algorithm developed by Yoshihara et al. [19], which computationally evaluates stromal and immune cell infiltration through systematic scoring of cell-type-specific gene expression signatures. The algorithm xCell [20] computes cell-type-specific enrichment scores by evaluating correlations between transcriptional profiles and cellular signatures. Both ESTIMATE and xCell algorithms have been widely employed across multiple cancer types, including lung adenocarcinoma [21,22], colon cancer [23,24], and adrenocortical carcinoma [25,26], demonstrating their robust versatility in oncological research.

In this study, we analyzed and calculated gene expression profiles from the GEO database by ESTIMATE and xCell and evaluated the prognostic impact of different scores on GBM patients. The immune cell subpopulations and immune cell subpopulations of the cohort were also analyzed by CIBERSORT. And we collect differentially expressed genes (DEGs) through these profiles, then some specific genes were identified by a series of statistical methods for prognostic analysis of patients, followed by a series of raw letter analyses, such as KEGG and mutation characteristics. As a result of these analyses, a deeper understanding of the potential clinical applications for prognostic stratification and treatment of GBM patients was provided.

## 2. Materials and Methods

### 2.1. The Data Collection and Pre-Processing

GBM-related gene expression profiles were obtained from public databases. From the GEO (https://www.ncbi.nlm.nih.gov/geo/, accessed on 31 March 2023) databases, we downloaded two cohorts, GSE16011 [27] and GSE7696 [28], for subsequent analysis. And in the TCGA (https://portal.gdc.cancer.gov, accessed on 22 June 2022) database, we downloaded the cohort named TCGA-GBM [29] of RNA-sequencing. All cohorts are mapped from probe IDs to gene symbols through the annotation files, and if there is a situation in which the gene symbol corresponds to multiple probes, the average value is selected as the final gene expression value. The total number of cases we finally collected was 753, of which GSE16011 (N = 155) was used as the training set, and GSE7696 (N = 80) and TCGA-GBM (N = 518) were used as the validation set.

### 2.2. Immune Infiltration Analysis

The relative or absolute abundance of immune and stromal cell populations in the samples was calculated using the three algorithms of xCell, ESTIMATE, and CIBERSORT [30]. Immune and mechanistic proportions were calculated for each patient using the R packages (version 4.4.0) “ESTIMATE” (version 1.0.13) and “xCell” (version 1.1.0), respectively, and the proportions of 22 immune cells in the tumor microenvironment of the samples were loaded and calculated via the CIBERSORT website (http://cibersort.stanford.edu/).

### 2.3. Data Processing and Acquirements of DEGs

Data were normalized and processed in the R environment (version 4.2.1, https://www.r-project.org/). The “limma” package (version 3.60.6) [31] was used for the acquisition of DEGs in the GSE16011 profile. The screening conditions for the DEGs were *p*-value < 0.05 and |log2FC| ≥ 2.

### 2.4. Construction of Risk Scoring Model

First, we divided the patients into high and low scores based on the estimated score. Then, the limma package was used to calculate the differential genes. Using the coxph function in the R package “survival ” (version 3.8.3), hazard ratio (HR) and *p*-values were calculated for each gene of the differential gene. Then, candidate genes with *p*-values less than 0.05 were used as input to the Least Absolute Shrinkage and Selection Operator (lasso) Cox regression model. The lasso regularization adds a penalty parameter (λ) to the Cox regression model, which allows some candidate genes to be ignored in the evaluation of the output, and 22 genes are left after the lasso regularization. Finally, RF was used for further screening, and we finally identified six genes for risk score (RS) model construction:

(1) $$RS=\sum_{i=1}^{N}(Expression_{i}\times Coefficient_{i})$$

The meanings of the parameters are as follows: where N is the number of genes, Expression is the gene expression values, and Coefficient is the Cox coefficients.

### 2.5. RS Prognostic Analysis

We divided patients into high and low groups using the R package “maxstat”(version 0.7.25) [32] to determine the optimal breakpoint for GBM. Kaplan–Meier survival curves were used to assess survival differences between the GBM patient groups. Multivariate Cox regression analysis was performed to evaluate the significance of each variable on the risk of survival. Time-dependent consistency index (C-index) and time-dependent receiver operating characteristic (tROC) analyses were performed using the R package “timeROC” (version 0.4) to compare the predictive power of survival across conditions.

### 2.6. Mutation Analysis

Mutation data for GBM cases were obtained from the TCGA database and stored in mutation annotation format (MAF) files, and a series of analyses of the mutation data were performed using the R package “maftools” (version 2.20.0) [33].

### 2.7. Enrichment Analysis

GO analysis was performed using the R package “Clusterprofiler” (version 4.12.6) for the three modules of biological processes, cellular localization, and molecular function, and KEGG for pathway enrichment. The corrected false discovery rate (FDR) as of standard <0.05 was considered significant.

### 2.8. Integrated Prognostic Model Construction and Validation

Based on the clinical information and trait genes used for risk scores in the selected data, clinical information and trait genes were evaluated using multivariate Cox proportional risk regression analysis, and nomogram scores were plotted using the R package “rms” (version 7.0.0), and we generated 1-, 3-, and 5-year calibration curves using a bootstrap method with 1000 resamples to validate the performance of the column line plots and to clearly show the ratio of the corresponding time points to the entire cohort.

### 2.9. External Data Validation

We used the external database Tumor Immune Estimation Resource (TIMER) (https://cistrome.shinyapps.io/timer/) to validate the differential expression of genes in different cancers used in the prognostic analysis model and to validate the associated immune infiltration. The Human Protein Atlas (HPA) (https://www.proteinatlas.org/) was used to validate protein expression of genes in normal and tumor tissues.

### 2.10. Data Analysis and Processing

GEO and TCGA data analysis was performed in the R environment (version 4.2.1). Kaplan–Meier and Cox analyses were performed using the R package “survival” (version 3.8.3), where log-rank tests and univariate Cox proportional risk regression generated *p*-values and HRs with 95% confidence intervals (CIs). The package “Clusterprofiler” (version 4.12.6) performs functional annotation. *p* < 0.05 was considered statistically significant.

## 3. Results

### 3.1. Database Queue Information

The study cohort comprised 155 GBM cases from GSE16011 (training set), 80 cases from GSE7696 (validation set), and 518 cases from TCGA. As presented in Table 1, the median patient age was 55 years. The training set included 105 male and 50 female patients. Given the aggressive nature and poor prognosis of GBM, mortality outcomes were observed in 147 cases, with only eight survivors in the training cohort.

### 3.2. Analysis Using ESTIMATE xCell and CIBERSORT

**Figure 1:**
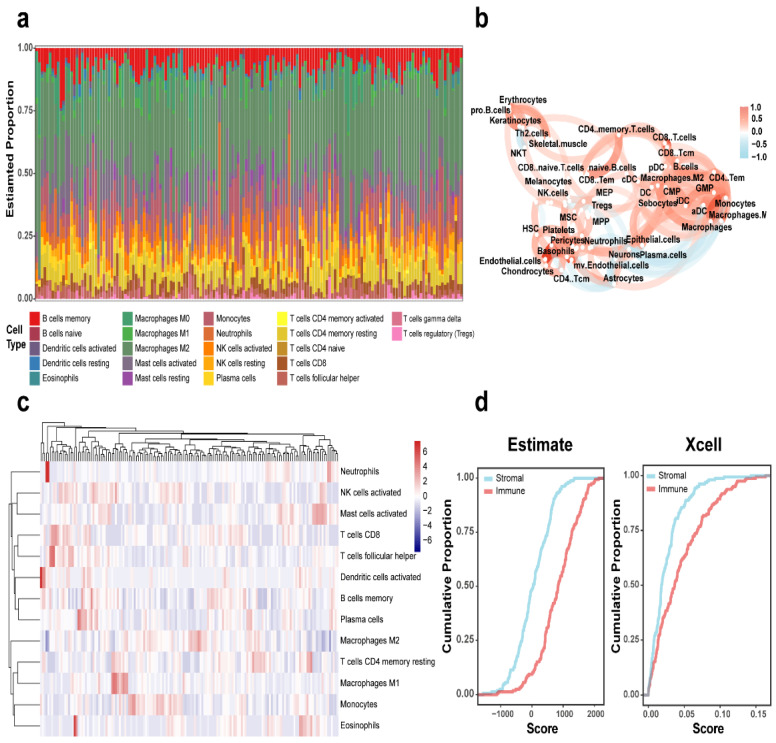
Immune landscape characterization in GBM. (**a**) CIBERSORT-derived histogram of 22 immune cell fractions in GBM. (**b**) Immune cell interaction network in GBM. (**c**) Heatmap depicting immune cell distribution across GBM cases. (**d**) Cumulative plots of ESTIMATE and xCell-calculated immune/stromal scores in the GBM cohort.

The CIBERSORT algorithm was employed for cellular characterization, revealing tumor-associated macrophages and T cells as the predominant immune cell populations within the tumor microenvironment (Figure 1a). Concurrently, immune cell infiltration levels were quantified using the xCell algorithm, with correlation network plots illustrating intercellular relationships among diverse immune cell types (Figure 1b). Figure 1c displays the corresponding immune infiltration correlation heatmap. Further analysis of 155 GSE16011 cases using both ESTIMATE and xCell algorithms demonstrated the relative abundance of immune versus stromal components in GBM patients (Figure 1d). The cumulative distribution curves of algorithm-derived scores consistently indicated significantly higher immune cell scores compared with stromal cells, underscoring the predominant role of immune infiltration in the GBM tumor microenvironment.

### 3.3. ESTIMATE Score and Overall Survival

**Figure 2:**
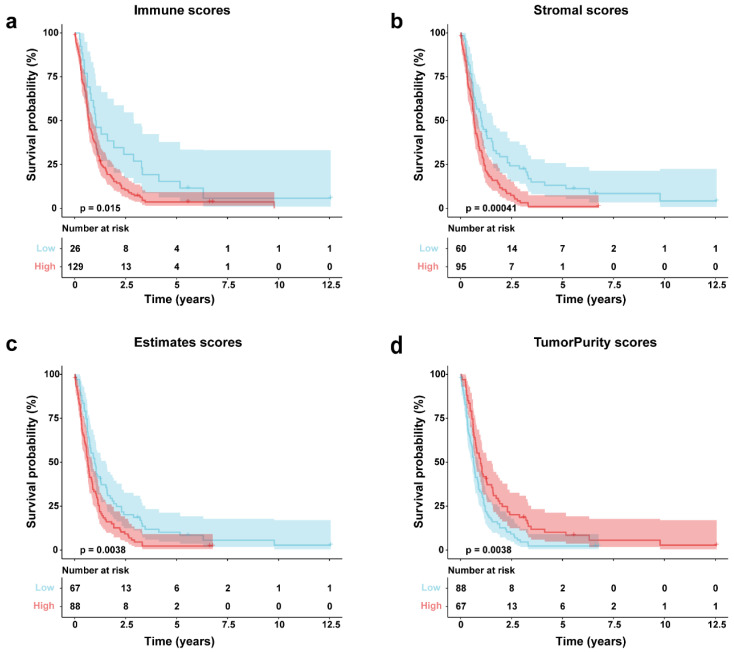
GSE16011 GBM survival analysis via Kaplan–Meier by score type. (**a**) Immune scores; (**b**) stromal; (**c**) estimates scores; (**d**) tumor purity scores.

Immune, stromal, and tumor purity scores were determined using ESTIMATE applied to the GSE16011 gene expression profile. Patients were then stratified into high- and low-score groups based on optimal cutoff points. Survival analysis revealed significant differences between these high- and low-scoring subgroups for all four scores. We observed an inverse relationship between overall survival time and immune (*p* = 0.0015), stromal (*p* = 0.0015), and ESTIMATE scores (*p* = 0.0038); essentially, lower scores were associated with a more favorable prognosis (Figure 2a–c). On the flip side, tumor purity scores showed a positive correlation with overall survival; put simply, higher scores indicated a better prognosis (*p* = 0.0038) (Figure 2d).

### 3.4. Establishment of Risk Score (RS) Model in GBM Patients

**Figure 3:**
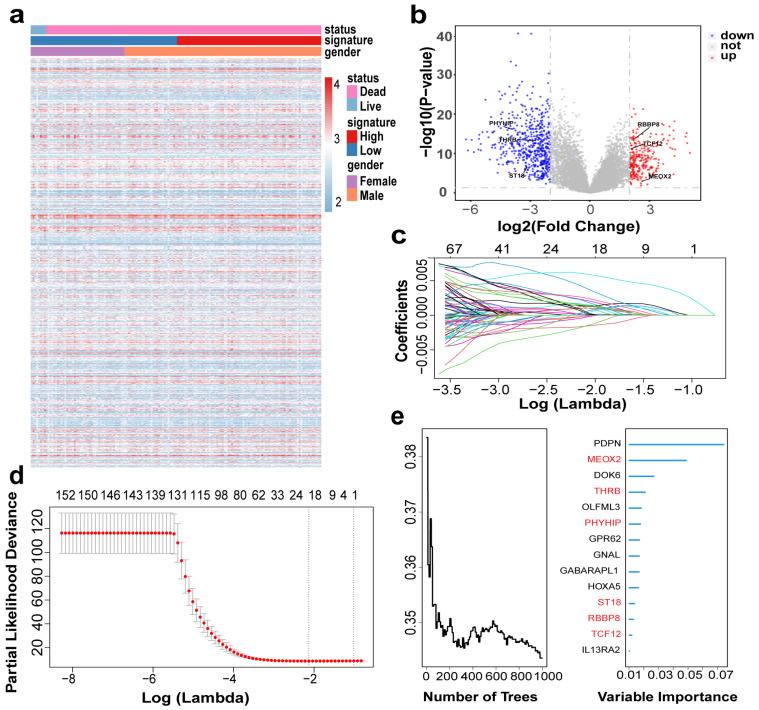
Construction of risk model based on GBM cohort: (**a**) Heatmap showing gene distribution and clinical information of the cohort; (**b**) Volcano plot showing differential gene expression. Up (red): upregulated genes (logFC > 2, *p*-value < 0.05); Down (blue): downregulated genes (logFC < −2, *p*-value < 0.05); Not (grey): non-significant genes. (**c**,**d**) Llasso Cox model analysis identified 22 genes. The different colors of the curves represent the changes in coefficients of different genes. (**e**) RF analysis (left) evaluated prediction error rates across decision trees to determine model convergence , while variable importance scores (right) identified the top 14 survival-associated genes (final selected *MEOX2, PHYHIP, RBBP8, ST18, TCF12*, and *THRB*, highlighted in red).

Based on the above studies, we evaluated the tumor microenvironment of GBM patients by ESTIMATE, xCell, and CIBERSORT and found a significant correlation between immune infiltration and GBM. We aimed to develop a model for patient prognosis assessment based on gene expression. Figure 3a shows the heat map information based on the training set, and the heat map gives us a preliminary understanding of their expression profile and clinical information. From the cumulative curves of the immune score and stromal score of ESTIMATE, we could obtain that immune infiltration has an important role in the GBM microenvironment (Figure 1d) and a low immune score has a better prognosis (Figure 2a), so we selected patients with low immune scores for the subsequent analysis. First, we analyzed differential genes in patients with low immune scores, including 263 “up” and 539 “down” genes (Figure 3b). The complete list of differentially expressed genes, including log2 fold changes and *p*-values for all identified transcripts, has been provided in Supplementary Table S1. Then we analyzed differential genes by univariate Cox, and the obtained genes were selected for optimal λ values for the lasso Cox regression model (10-fold cross-validation) to obtain 22 genes (Figure 3c,d), and then RF analysis was performed to obtain 14 genes (Figure 3e). Finally, six genes (*MEOX2, PHYHIP, RBBP8, ST18, TCF12*, and *THRB*) were identified by permuting the obtained genes to construct the risk assessment model:

(2) RS=(0.58×MEOX2)+(0.35×PHYHIP)+(0.57×RBBP8)                            +(0.36×ST18)+(−
0.67×TCF12)+(0.35×THRB)

**Figure 4:**
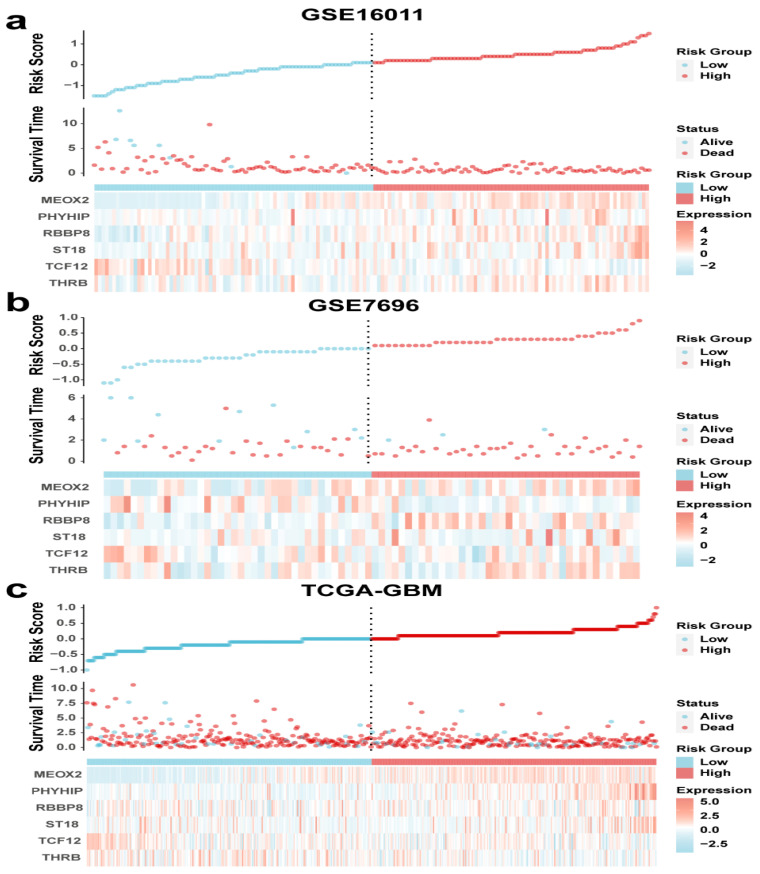
Risk score curves, survival time and survival status scatter plots, and clustering heat maps for (**a**) GSE16011, (**b**) GSE7696, and (**c**) TCGA-GBM.

Based on the six-gene signature screened, we calculated the risk scores for the training set and validation set cohorts separately and made correlation heat maps. As seen in Figure 4a, the higher the risk score in the training set, the higher the expression of *MEOX2, PHYHIP, RBBP8, ST18*, and *THRB*, and the lower the expression of *TCF12*. The results of the validation set and the training set were basically consistent (Figure 4b,c). The results of Cox analysis also showed that *TCF12* was lowly expressed in the high-risk score group, indicating that it may be a protective factor (Table 2).

### 3.5. Six-Gene Risk Score Model Prognostic Analysis

**Figure 5:**
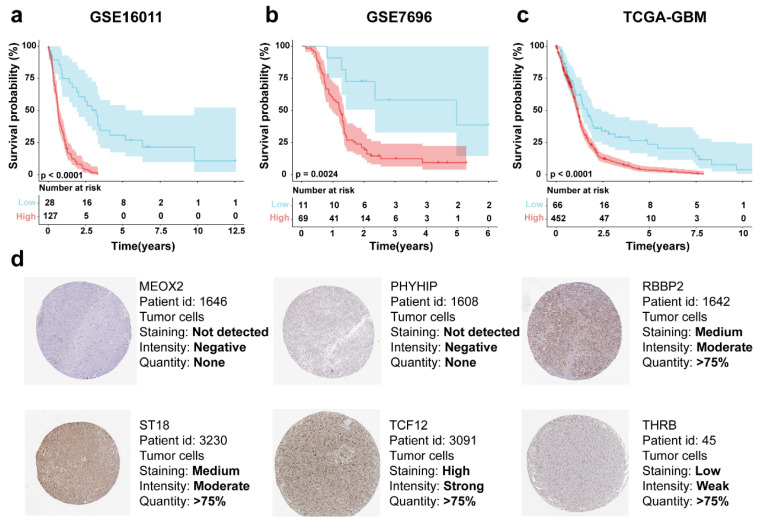
Risk score-based KM survival curves for the patient cohorts (**a**) GSE16011, (**b**) GSE7696, and (**c**) TCGA-GBM. (**d**) Protein expression of *MEOX2, PHYHIP, RBBP8, ST18, TCF12,* and *THRB* in tumor tissues in the HPA database.

Patients were stratified into high- and low-risk groups based on optimal cutoff values derived from the six-gene signature risk score. Kaplan–Meier analysis confirmed significantly improved survival in low-risk patients within the GSE16011 training cohort (*p* < 0.01, Figure 5a). Univariable and multivariable Cox analyses were done for age, gender, and gene signature, respectively. From the results of GSE16011 analysis in Table A1, it was shown that the univariable Cox for age (HR = 2.72, 95% CI = 1.92–3.86, *p* < 0.01) and gene signature (HR = 2.47, 95% CI = 1.73–3.51, *p* < 0.01) were significant, and both were potential risk factors, while the multivariable Cox analysis also showed that age (HR = 2.36, 95% CI = 1.65–3.38, *p* < 0.01) and gene signature could be used as risk factors (HR = 2.15, 95% CI = 1.50–3.10, *p* < 0.01). The validation set GSE7696 and TCGA univariable Cox for age (GSE7696, *p* < 0.01; TCGA, *p* < 0.01) and gene signature (GSE7696, *p* < 0.01; TCGA, *p* < 0.01) were significant, and the clinical information of gender in all three datasets did not reflect significance, and it can be obtained that gender is not a risk factor.

In Table 3, the relationship between gene signature and clinical information of patients was verified by the Chi-square test, and there was a significant correlation (*p* < 0.01) between gene signature and age in GSE16011, and no significant correlation with gender. The GSE7696 gene signature as the validation set had no significant correlation with clinical information, while the gene signature in TCGA had a significant correlation with age (*p* < 0.01) and no significant correlation with gender.

Representative protein expression of MEOX2, PHYHIP, RBBP8, ST18, TCF12*,* and THRB in tumor tissues was demonstrated based on immunohistochemical analysis in the HPA database. MEOX2 and PHYHIP, showed no significant protein expression in tumor tissues; the THRB showed “low intensity”, RBBP8 and ST18 showed “medium intensity”, and, interestingly, the TCF12 showed “high intensity” (Figure 5d).

### 3.6. Integrated Prognostic Model with Gene and Clinical Information

**Figure 6:**
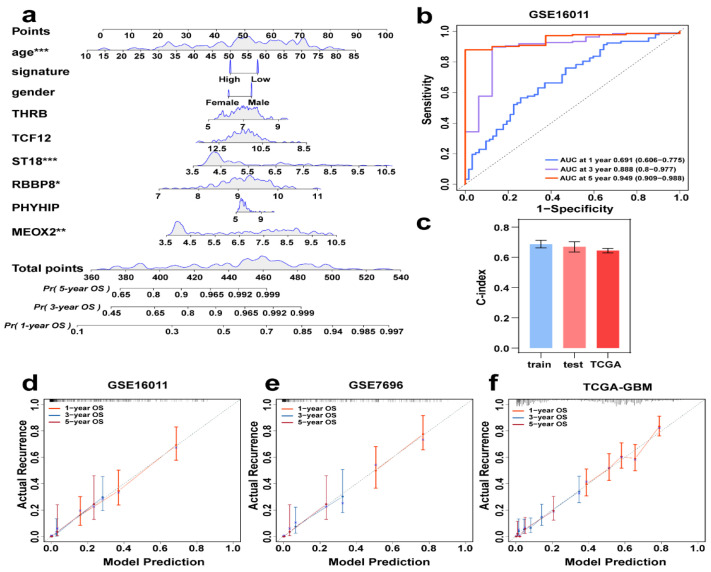
Individual integrated prognostic model construction. (**a**) A prognostic nomogram consisting of clinical information and genes together, predicting survival by score for different years. * *p *< 0.05, ** *p *< 0.01, *** *p* < 0.001***.*** (**b**) GSE16011Time-ROC curves of 1, 3, and 5 years AUC. (**c**) C-index values of training (GSE16011), test (GSE7696), and TCGA data. (**d**–**f**) Calibration curves of GSE16011, GSE7696, and TCGA-GBM, where the red, blue, and purple lines are the one-year, three-year, and five-year projections, respectively.

Although a single risk score is beneficial to patient prediction analysis, clinical information and the genes screened in this study also have an important impact on patient prognosis, so we created a nomogram that combines signature, clinical information (sex and age), and multiple parameters of genes (*MEOX2, PHYHIP, RBBP8, ST18, TCF12,* and *THRB*) to predict the probability of 1-year, 3-year, and 5-year overall survival (Figure 6a). In Figure 6b, the time-AUC values for the training set GSE16011 are shown, where the 1-year (0.755, 95% CI: 0.606–0.775), 3-year (0.888, 95% CI: 0.800–0.997), and 5-year (0.949, 95% CI: 0.909–0.988) AUC values demonstrate the excellent prediction confidence of our model. Figure 6c shows the C-index values of the training and validation sets (GSE16011: 0.69, GSE7696: 0.67, TCGA-GBM: 0.64), and it can be seen from the C-index values that our model has good stability. Figure 6d shows the calibration curve of the training set, which proves that our nomogram has a good predictive property by comparing the true and predicted values, and then the calibration curves of the two validation sets, GSE7696 and TCGA-GBM, were done separately (Figure 6e,f), which also prove the excellent performance of the model.

### 3.7. Enrichment Analysis of DEGs

**Figure 7:**
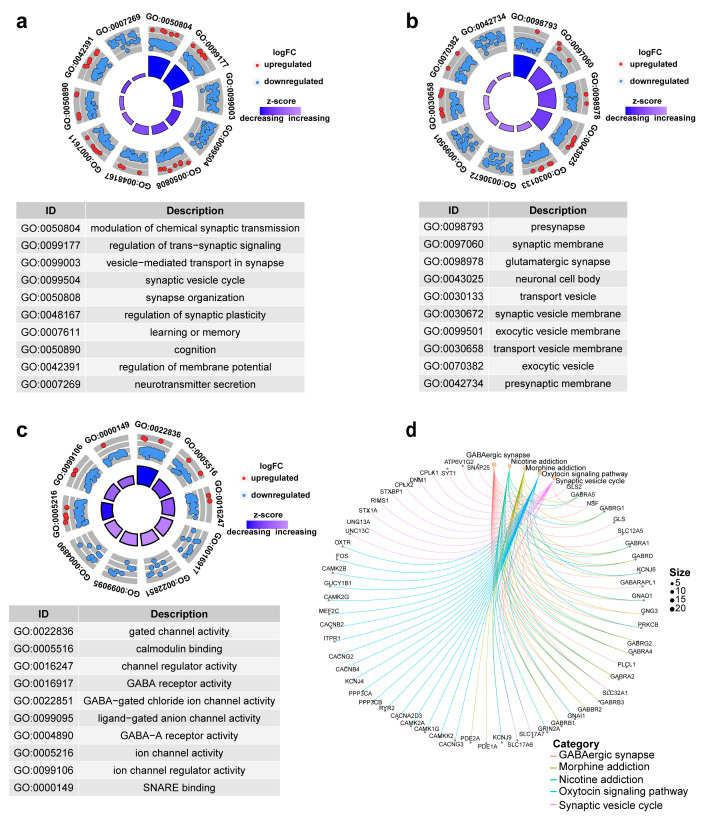
Top 10 significantly enriched functional terms. (**a**) biological process (BP) (**b**) cellular component (CC) (**c**) molecular functions (MFs) of GO analysis. (**d**) KEGG-Gene-Concept Network of top five categories.

To elucidate the biological significance of differentially expressed genes (DEGs), we conducted GO and KEGG pathway analyses. Figure 7a–c respectively displays the top 10 enriched terms in biological processes (BPs), cellular components (CCs), and molecular functions (MFs). Among them, modulation of chemical synaptic transmission, presynapse, and gated channel activity are the most noteworthy results. Figure 7d is the KEGG-Gene-Concept Network, showing the top five major pathways and the genes associated with them, which can lead to more DEGs associated with the GABAergic synapse pathway.

### 3.8. GBM Mutation Analysis

**Figure 8:**
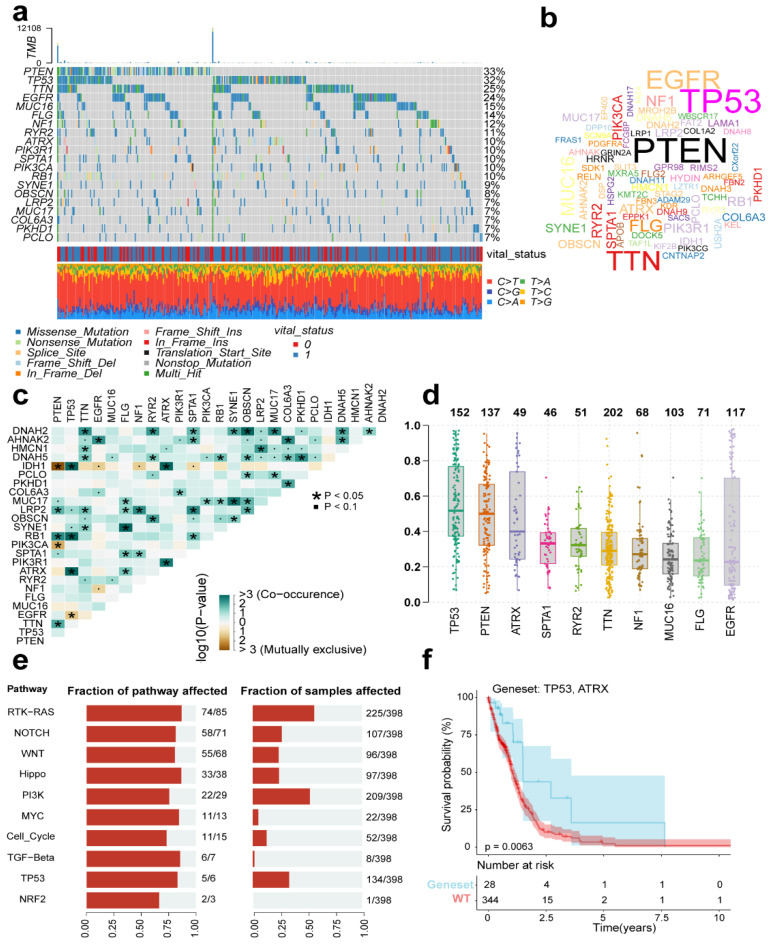
Mutational profiles across TCGA-GBM cases. (**a**) Oncoplot displaying the top 20 most recurrently mutated genes per variant class. (**b**) The gene cloud shows the total number of samples of each mutated gene as a proportion of the total number of mutations, and the size of each gene is proportional to the total number of samples in which it is mutated. (**c**) The correlation graph shows the mutually exclusive or coexisting status of the most mutated top genes. The green color is for mutated genes that tend to coexist; the yellow color is for mutually exclusive genes, and the shade of color indicates the significance. (**d**) Variant allele frequency (VAF) distribution in mutants (box plot). (**e**) Top 10 significantly enriched oncogenic pathways with sample prevalence rates. (**f**) Prognostic impact of mutant gene sets (KM analysis).

To explore mutational associations with the tumor immune microenvironment (TIME), we systematically evaluated TCGA-GBM genomic data, identifying the 20 most recurrently mutated genes across the cohort. The oncoplot identified four genes (PTEN, TP53, TTN, EGFR) with mutation frequencies exceeding 20% (Figure 8a). We also plot the gene cloud, where the size of each gene is proportional to the total number of samples in which it is mutated, and it is also evident that four genes have the highest mutation frequency in the sample (Figure 8b). Alongside single mutations, we also looked for co-occurring mutations within the top 20 mutated genes using a pairwise Fisher’s exact test. The results, visualized in Figure 8c, highlight genes that tend to hang out together (shown in green) and those that tend to be mutually exclusive (shown in yellow). The color intensity reflects the statistical significance, giving us a potential rationale for clinical strategies. Shifting gears to Figure 8d, we see the variant allele frequency (VAF), which is a handy tool for gauging tumor heterogeneity and purity. Plus, whether the VAF is high or low might just have a bearing on how the cancer progresses. When we dug into oncogenic pathways, RTK-RAS, PI3K, and TP53 popped up as the main players in these cases, as you can see in Figure 8e. Lastly, we zeroed in on the mutated genes that seemed to have the biggest impact on prognosis—namely TP53 and ATRX. What’s interesting is that this particular set of mutated genes was associated with a favorable outcome (Figure 8f).

### 3.9. External Validation of Immune Infiltration and Gene Expression

**Figure 9:**
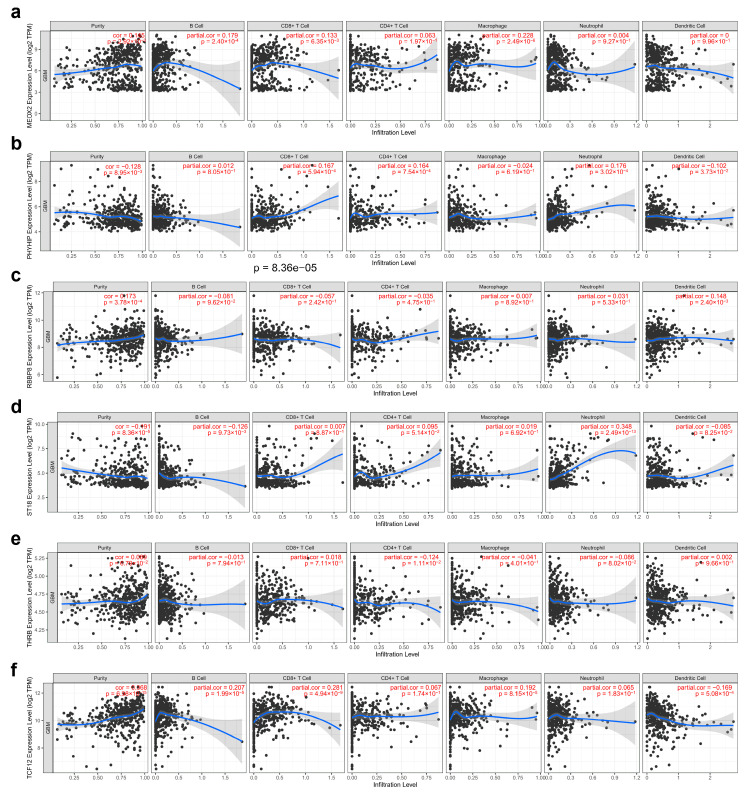
Correlation analysis of gene tagging with tumor purity and immune cell infiltration in GBM cancer: (**a**) *MEOX2,* (**b**) *PHYHIP,* (**c**) *RBBP8,* (**d**) *ST18,* (**e**) *TCF12,* and (**f**) *THRB*. Each black dot represents a case.

We analyzed the potential associations between immune cell infiltration and GBM prognosis-related genes (*MEOX2, PHYHIP, RBBP8, ST18, TCF12,* and *THRB*) through the online database TIMER, where most genes were significantly associated with purity, containing *MEOX2, PHYHIP, RBBP8, ST18,* and *TCF12*, and conversely, the fewest genes were significantly associated with macrophage (*MEOX2, TCF12*) and neutrophil (*PHYHIP, ST18*) (Figure 9).

**Figure 10:**
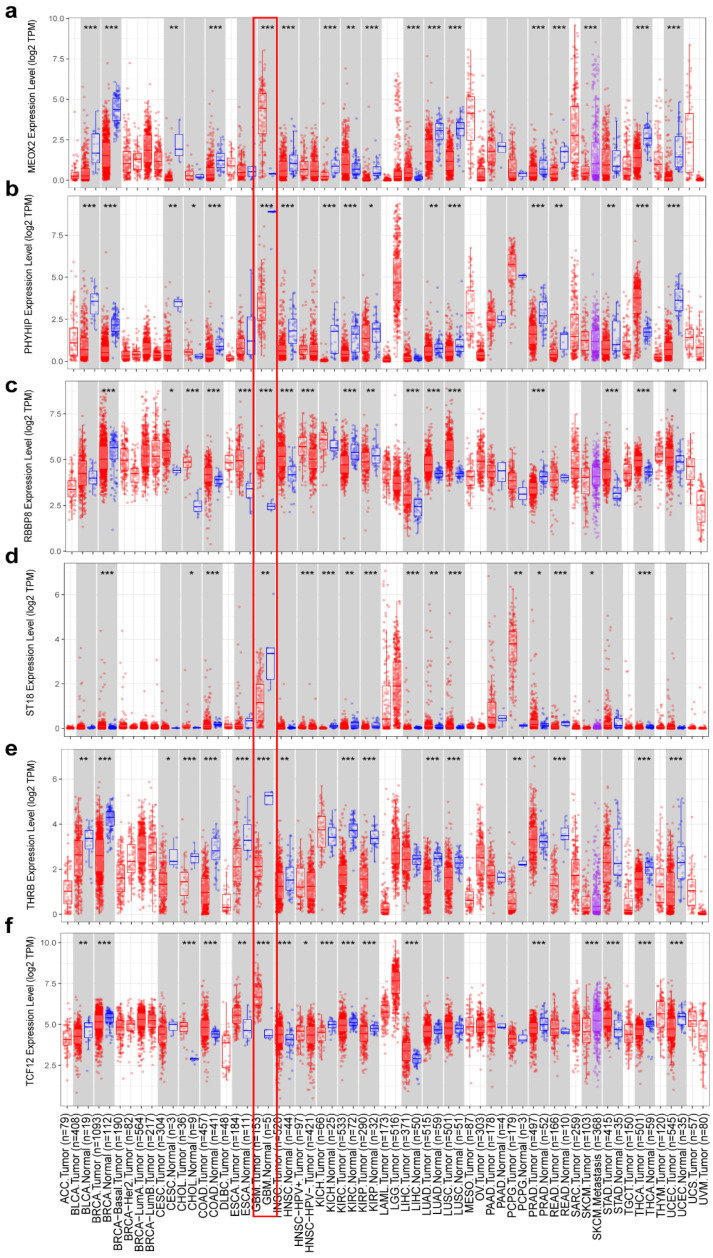
Differential expression of genes between normal and cancerous tissues (**a**) *MEOX2,* (**b**) *PHYHIP,* (**c**) *RBBP8,* (**d**) *ST18,* (**e**) *TCF12,* and (**f**) *THRB*. The red rectangular box shows the gene expression in GBM tumor tissue and normal tissue. * *p* < 0.05, ** *p* < 0.01, *** *p* < 0.001.

Consistent with the differential gene expression patterns observed in Figure 3b, our analysis of TCGA data in TIMER further validated that *MEOX2* (Figure 10a), *RBBP8* (Figure 10c), and *TCF12* (Figure 10f) were significantly upregulated in tumor tissues, while *PHYHIP* (Figure 10b), *ST18* (Figure 10d), and *THRB* (Figure 10e) showed marked downregulation. This concordance between our experimental results (Figure 3b) and large-scale TCGA database analysis reinforces the reliability of these candidate genes as tumor-associated markers.

## 4. Discussion

Gliomas are primary malignant tumors characterized by high aggressiveness. Beyond traditional treatments such as surgery, chemotherapy, and radiotherapy, increasing attention has been paid to the immunological aspects of glioma progression. Immune evasion is recognized as a hallmark of cancer, not only involving intrinsic changes in tumor cells but also alterations to the surrounding tumor microenvironment that reduce immune cell recognition. Therefore, understanding glioma requires not only investigating tumor-intrinsic molecular features but also analyzing the associated immune landscape [34,35].

In this study, we characterized the GBM TME by estimating the abundance of 22 immune cell types using CIBERSORT. Immune cell correlations were further assessed by Xcell, while ESTIMATE was applied to compute immune, stromal, tumor purity, and overall ESTIMATE scores. Cumulative distribution analyses revealed that immune scores were generally higher than stromal scores, suggesting a dominant role of immune infiltration. Based on optimal cutoffs of these scores, samples were stratified into high and low groups, and downstream analysis was performed focusing on the immune score dimension.

To identify molecular features associated with the GBM immune microenvironment, we first did differential gene analysis for patients with low immune scores and then combined univariate Cox, lasso regression and RF to identify prognosis-related gene signatures (*MEOX2, PHYHIP, RBBP8, ST18, TCF12,* and *THRB*) to establish a risk score approach for GBM patients, followed by multifactorial Cox analysis with clinical information, and risk scores still had significant prognostic power. Further analysis of its functional enrichment and mutations was performed.

Genes of interest in the gene signature established in this study have been studied in the prognosis of a variety of cancers. For example, in a tumor context, Zhou et al. reported that decreased expression of *MEOX2* in hepatocellular carcinoma caused a decrease in overall survival [36], and the same phenomenon was found by Tian in a study of the *MEOX2* gene in laryngeal cancer [37]. And Avila-Moreno et al. also found that *MEOX2* expression affects chemotherapy resistance in lung cancer studies [38]. And in our study, high expression of *MEOX2* was detrimental to the prognosis of patients. Miyako Yamamoto experimentally demonstrated that *PHYHIP* is expressed at lower levels in breast cancer cell lines than in normal cells and that this gene may have an important relationship with breast cancer [39]. Li et al. also established *PHYHIP* as a prognostic model gene during their study on the prognosis of gastric cancer and found that high expression of *PHYHIP* leads to a lower prognostic survival time [40]. In our study, *PHYHIP* was also used as a prognostic model for GBM cancers.

The *RBBP8* gene has also been extensively studied. In a study of plasma cell myeloma, Zhang et al. found that high expression of RBBP8 was significantly associated with low patient survival [41]. Yu et al. found experimentally that overexpression of *RBBP8* in gastric cancer could promote the G1/S transition in GC cells by inhibiting P21 levels [42]. Yu et al. found frequent mutations of prognosis-related genes such as *ST18* in residual tumors in small cell lung cancer, possibly by affecting chemotherapy resistance and influencing the prognosis of SCLC patients [43]. Methylation of *ST18* in a study of head and neck cancer (HNC) by Ribeiro was also found to have a prognostic effect on patients [44].

In their study, Labreche et al. showed that in mesenchymal oligodendroglioma, *TCF12* produces mutations that impair *TCF12* transcriptional activity and that this correlates with more aggressive tumors [45]. Interestingly, in our study, *TCF12* was found to be a protective factor in GBM. *THRB* is associated with prognosis in various cancer entities and has been used in previous studies as a prognostic marker in cancers such as lung adenocarcinoma, high-grade plasmacytoma, ovarian cancer, and colorectal cancer [46,47,48]. It has demonstrated its potential as a therapeutic target for cancer. High expression of *THRB* in this study resulted in poor prognosis in GBM patients.

Subsequently, we co-constructed prognosis-related genes and clinical information to construct columnar maps for predicting patients’ survival probabilities at 1, 3, and 5 years. It was also validated by calibration curves and found good prediction results in both training and test sets. Finally, we also performed validation using external datasets. Using the TIMER database, we found that the prognosis-related genes identified in this study were all differentially expressed in cancer tissues. The correlation of prognosis-related genes with immune infiltration was also explored. It was found that the most genes were significantly associated with purity and, conversely, the fewest genes were significantly associated with macrophages and neutrophils. The expression of the proteins encoded by the different genes was demonstrated by immunohistochemistry in the HPA database.

However, there are some limitations in this study. First, we used case cohort data from a database to analyze and calculate the genetic labels, which need to be validated by clinical data to prove their validity. In addition, we only performed a follow-up analysis based on their immune scores, but the tumor microenvironment is a complex environment, and the immune scores alone cannot reveal the complete role of the tumor microenvironment, which needs to be analyzed from more aspects and integrated with the results of each stratum to understand the overall impact of the tumor microenvironment on patient prognosis.

## 5. Conclusions

This study establishes a six-gene prognostic signature based on the immune landscape of the GBM TME. By integrating computational biology and machine learning techniques, we identified *MEOX2, PHYHIP, RBBP8, ST18, TCF12,* and *THRB* as key molecular markers associated with GBM patient outcomes. The signature shows strong performance in stratifying patient survival risk and provides insights into the biological mechanisms linking immune context and tumor progression. These findings enhance our understanding of microenvironment-driven heterogeneity in GBM and offer a foundation for future research on targeted interventions and tumor–immune interactions.

## Figures and Tables

**Table 1:** Clinical information of the Gene Expression Omnibus (GEO) and The Cancer Genome Atlas (TCGA) datasets.

Characteristic	GSE16011	GSE7696	TCGA
Age (years)			
>55	78	28	306
≤55	77	52	212
Sex			
Female	50	21	204
Male	105	59	314
Vital status			
Alive	8	39	77
Dead	147	42	441

**Table 2:** Prognosis of the four genes in the signature.

ENSEMBL ID	Symbol ID	Gene Name	Coef	*p*-Value	Prognostic Indicator
ENSG00000106511	*MEOX2*	Mesenchyme Homeobox 2	0.58	<0.01	high
ENSG00000168490	*PHYHIP*	Phytanoyl-CoA 2-Hydroxylase Interacting Protein	0.35	<0.01	high
ENSG00000101773	*RBBP8*	RB Binding Protein 8	0.57	<0.01	high
ENSG00000147488	*ST18*	Suppression Of Tumorigenicity 18	0.36	=0.03	high
ENSG00000140262	*TCF12*	Transcription Factor 12	−0.67	<0.01	low
ENSG00000151090	*THRB*	Thyroid Hormone Receptor Beta	0.35	=0.04	high

**Table 3:** The IRG signature and clinical characteristics Chi-square table in GBM patients.

Variables	Status	Low	High	*p*
GSE16011 dataset (N = 155)				
Age				<0.01
	≤55	48	29	
	>55	30	48	
Gender				0.36
	Female	22	28	
	Male	56	49	
GSE7696 dataset (N = 80)				
Age				0.10
	≤55	30	22	
	>55	10	18	
Gender				0.31
	Female	8	13	
	Male	32	27	
TCGA dataset (N = 518)				
Age				<0.01
	≤55	122	90	
	>55	137	169	
Gender				1
	Female	102	102	
	Male	157	157	

## Data Availability

The datasets for this study can be found in the GEO databases (https://www.ncbi.nlm.nih.gov/geo/, accessed on 31 March 2023) and the TCGA databases (https://portal.gdc.cancer.gov, accessed 22 June 2022).

## Supplementary Materials

The following supporting information can be downloaded at: https://www.mdpi.com/article/10.3390/genes16080861/s1, Table S1: Complete list of differentially expressed genes.

## Abbreviations

The following abbreviations are used in this manuscript:

CNS	central nervous system
GBM	Glioblastoma
TME	tumor microenvironment
KM	Kaplan–Meier
RF	Random forest
TCGA	The Cancer Genome Atlas
GEO	Gene Expression Omnibus

## Appendix A

**Table A1:** Cox regression analysis of the IRG signature and clinical information with GBM survival.

Variables		Univariable Cox				Multivariable Cox			
		HR	95% CI of HR		*p*	HR	95% CI of HR		*p*
			Right	Left			Right	Left	
GSE16011 (N = 155)									
Age	>55 vs. ≤55	2.72	1.92	3.86	<0.01	2.36	1.65	3.38	<0.01
Sex	Male vs. female	1.11	0.78	1.57	0.56	1.23	0.87	1.74	0.24
Signature	High risk vs. low risk	2.47	1.73	3.51	<0.01	2.15	1.50	3.10	<0.01
GSE7696 (N = 80)									
Age	>55 vs. ≤55	1.97	1.18	3.29	<0.01	1.55	0.89	2.70	0.11
Sex	Male vs. female	0.92	0.53	1.61	0.77	1.05	0.60	1.84	0.86
Signature	High risk vs. low risk	2.27	1.37	3.76	<0.01	1.97	1.14	3.39	0.01
TCGA (N = 518)									
Age	>55 vs. ≤55	2.00	1.64	2.44	<0.01	1.94	1.58	2.37	<0.01
Sex	Male vs. female	1.16	0.96	1.41	0.13	1.12	0.92	1.36	0.25
Signature	High risk vs. low risk	1.30	1.07	1.57	<0.01	1.17	0.97	1.42	0.11
